# Serum Uric Acid Levels and the Risk of Impaired Fasting Glucose: A Prospective Study in Adults of North China

**DOI:** 10.1371/journal.pone.0084712

**Published:** 2013-12-23

**Authors:** Yeqiang Liu, Cheng Jin, Aijun Xing, Xiurong Liu, Shuohua Chen, Dongqing Li, Ping Feng, Jinquan Liu, Zhiguo Li, Shouling Wu

**Affiliations:** 1 The General Hospital of Tianjin Medical University, Tianjin, China; 2 The Kailuan Hospital, Hebei United University, Tangshan, China; 3 The Hebei United University, Tangshan, China; University of Catanzaro Magna Graecia, Italy

## Abstract

**Objective:**

To prospectively investigate the association between serum uric acid (SUA) level and incidence of impaired fasting glucose (IFG) in adult Chinese.

**Methods:**

We evaluated 13,328 women and 41,350 men without diabetes and IFG. The participants were classified into quintile according to baseline level of SUA. Data were analyzed to examine the association between SUA levels and the incidence of IFG. We used Cox regression models to estimate the relative risk of IFG after adjusting for known risk factors.

**Results:**

For men, the second quintile of SUA has the lowest cumulative incidence of IFG (29.9%); the fifth quintile of SUA has the highest cumulative incidence of IFG (35.6%). After corrected with Cox regression, the first quartile and the fourth quartile have higher cumulative incidence of IFG than the second quintile, with the HR of 1.11(1.05-1.17) and 1.07(1.01-1.13), respectively. For women, the first quartile of SUA has the lowest cumulative incidence of IFG (20.7%), while the fifth quintile of SUA has the highest cumulative incidence of IFG (30.0%). However, there is no significant difference in IFG between different quintile after adjusted with Cox regression.

**Conclusions:**

The results of this prospective study suggest that there is a higher risk of developing IFG in association with low or high SUA concentrations for men. These relationships were independent of other known risk factors. There is no significant correlation in the risk of developing IFG in association with SUA concentrations for women. Analyses excluding participants with hypertension or with hyperlipidemia and analyses with participants stratified by age reached similar conclusion.

## Introduction

SUA is the product of purine metabolism. Hyperuricemia causes gout and is involved in other pathologic conditions. Previous studies found that level of SUA is associated with hypertension, coronary artery heart disease, metabolic syndrome, and abnormal glucose metabolism. It was demonstrated that hyperuricemia can cause through insulin resistance, enhanced oxidative stress, endothelia dysfunction, activation of the rennin-angiotensin system [1-4]. However, there are contradictions about the association between hyperuricemia and development of aforementioned diseases. Therefore, more investigations are needed to understand whether serum uric acid levels are causally involved in cardiovascular diseases or abnormal glucose metabolism [5,6]. With increasing numbers of people with diabetes or pre-diabetes in China [7], as an important component of the primary prevention in diabetes, it becomes more and more important to find the risk factors associated with abnormal glucose metabolism [8]. Previous studies showed that in adults elevated SUA is associated with diabetes [9-13], while others found no clear association between high SUA and abnormal glucose metabolism [6,14]. Conversely, it has been reported that serum uric acid was lower in people with diabetes than in normoglycaemic people [15,16]. The contradiction in conclusions may be due to that different populations were surveyed or different methods of survey were employed. Here, we carried out a 4 year perspective cohort study based on the data from Kailuan Study (Chinese Clinical Trial Registry number: ChiCTR-TNRC-11001489). The purpose of our study is to examine the association between serum uric acid and incidence of impaired fasting glucose (IFG) level in adults.

## Methods

### Ethics Statement

Our study was approved by the Ethics Committee of Kailuan General Hospital, in compliance with the Declaration of Helsinki. All participants or their legal representatives (for participants with dementia or illiteracy) signed informed consent forms (ICFs).

### Study participants

The data were obtained from health examinations of employees of the Kailuan Group LLC in Tangshan city in the central north of China, which is situated 150 km southeast of Beijing. The Kailuan community is a functional and comprehensive community owned and managed by the Kailuan Group. The Kailuan study was a prospective population-based cohort study in the Kailuan community. This study was designed to investigate risk factors for chronic diseases. All residents in the Kailuan community have routine medical examinations, which include physical examination, and routine blood, urine, and biochemical tests every two years without charge. Details of the Kailuan prospective study have been previously described [17-19,30]. From June 2006 to September 2007, a total of 101,510 (81,110 men, 20,400 women; aged 18-98 year old) employees and retirees agreed to participate, were enrolled after written informed consent obtained. They underwent the baseline survey regarding health status and lifestyle. All data collection was performed by trained doctors and nurses. 

Participants with a history of diabetes, or fasting blood glucose (FBG) ≥5.6mmol/L (n=30489), myocardial infarction (MI, n=1305), stroke (n=2336), cancer (n=327), or severe nephropathy (estimated Glomerular Filtration Rate; eGFR) ≤30 ml/min/1.73 m^2^, n=1043), or those with missing measurement of SUA and other measurements (n=1284) and those with missing data of 2008 or 2010(n=8439), were excluded, leaving 56287 participants. In the follow up study, 266 women and 1343 men were diagnosed as diabetes and thus were excluded. Therefore, the total participants of this study were 54678. The participants of this study have lower proportion of males and are younger in age compared to general population of Kailuan community. The participants also have lower systolic blood pressure (SBP), diastolic blood pressure (DBP), body mass index (BMI), triglycerides (TG), total cholesterol (TC), low-density lipoprotein cholesterol (LDL-C), and FBG. The participants also have lower proportion of smoking, alcohol drinking, history of hypertension or hyperlipidemia.

### Laboratory assessment

Blood samples were obtained from the antecubital vein and transfused into vacuum tubes containing EDTA in the morning after an overnight fasting period. Within 30 minutes of collection, the blood was centrifuged for 10 minutes at 3000 rotations per minute at 25 °C. An auto analyzer (Hitachi 747; Hitachi, Tokyo, Japan) was used to measure SUA (uric acid enzymatic method), FBG, serum creatinine, TC, TG, high-density lipoprotein cholesterol (HDL-C), LDL-C, and hypersensitive C-reactive protein (hs-CRP) at the central laboratory of Kailuan hospital. Kit was provided by the Biology Institute of North China. The coefficient of variations of SUA assessment was ≤6.0% for both within and between groups. The CKD-EPI Equation was used to estimate estimated glomerular filtration rate (eGFR) expressed in ml/min/1.73 m^2^ [20].

### Assessment of diabetes and IFG

Diabetes and IFG were defined by the criteria of American Diabetes Association. Diabetes was defined as follows: FBG≥7.0mmol/L and/or validated physician diagnosis and/or current use of glucose-lowering medications; IFG was defined as FBG ≥5.6mmol/L and ≤7.0mmol/L but excluding diabetes.

### Questionnaire assessment

Questionnaires were administered in person by research doctors. Smoking status and drinking status were classified using self-reported information as “never, former, or current.” The existence of preexisting stroke, MI, cancer, hyperlipidemia, or hypertension, was defined as any self-reported previous physician diagnosis. The use of antihypertensive, antihyperlipidemia, and glucose-lowering medications within the past 2 weeks of the baseline interview was self-reported.

### Anthropometric measurements and blood pressure measurement

Anthropometric indices included height and weight. All individuals were measured while they were wearing scrubs. Height was measured to the nearest 0.1 cm using a tape measure, and weight was measured to the nearest 0.1 kg using calibrated platform scales. BMI was calculated as body weight (kg) divided by the square of height (m^2^). Blood pressure (BP) was measured in the right arm with study participants in a sitting position using a regular mercury sphygmomanometer after resting for 15 minutes. Three consecutive BP readings were used to generate a mean value. Hypertension was defined as a mean DBP ≥90mmHg or a mean SBP ≥140mmHg or the use of antihypertensive medication indicated to treat high blood pressure.

### Statistical analysis

Data were input by data entry clerk of individual hospitals and transferred to Oracle10g database located in Kailuan Hospital through Internet. The data were analyzed using SAS 9.2. Continuous variables are presented as means ± standard deviation and compared using one-way ANOVA. Categorical variables are presented as n (%) and compared using x^2^ test. According to the baseline level of SUA, participants were assigned to quintile with the second quintile set as control. Cox proportional hazards regression was used to calculate hazard ratios. The data were adjusted first for age (Model 1), and then further for SBP, DBP, BMI, TG, TC, HDL-C, LDL-C, FBG, hs-CRP, history of hypertension, anti-hypertension medicine, history of hyperlipidemia, and hyperlipidermia medicine (Model 2). Based on model 2, the data were additionally adjusted for smoking, alcohol drinking (Model 3). Since there are interactions (p=0.002) in the effects of gender and serum uric acid on the new onset of impaired fasting glucose, male and female participants were analyzed separately. For all comparisons, the level of statistical significance was set at P < 0.05.

## Results

As shown in Table 1, compared to the quintile of lower SUA, the participants in the quintile with higher SUA are older in age, and higher in SBP, DBP, TG, TC, CRP, and BMI. The participants with higher SUA also have history of hypertension and hyperlipidemia, with higher percentage of smoking and alcohol drinking. During the follow-up period (3.6±0.9 years for women, 3.4±1.0 years for men), 3215 women and 13551 men developed IFG. For women, the first quartile of SUA has the lowest cumulative incidence of IFG (20.7%), while the fifth quintile of SUA has the highest cumulative incidence of IFG (30.0%). However, there is no significant difference in IFG between different quintile after adjusted with Cox regression. For men, the second quintile of SUA has the lowest cumulative incidence of IFG (29.9%); the fifth quintile of SUA has the highest cumulative incidence of IFG (35.6%). The cumulative incidence of IFG for the first and the forth quintile are 32.2% and 33.9%, respectively, which are slightly lower than the fifth quintile. After corrected with Cox regression, the first quartile and the fourth quartile have higher cumulative incidence of IFG than quintile 2, with the HR of 1.11(1.05-1.17) and 1.07(1.01-1.13), respectively (Table 2).

**Table 1:** Baseline characteristics of the population by gender and quintile of uric acid in the Kailuan Study in 2006-2010.

	Uric acid quintile				
	Quintile 1	Quintile 2	Quintile3	Quintile4	Quintile5
**Women**					
No. of patient	2644	2682	2659	2639	2704
Uric acid, µmol/L	<186.6	187.0-217.9	218.0-248.9	249.0-289.0	>289.0
Uric acid Median, µmol/L	162.0	203.0	232.0	267.0	324.5
Age, year	45.3±10.1	45.2±10.3	46.0±10.8	47.3±11.1	50.3±12.8
SBP, mmHg	118.0±18.3	119.0±18.5	121.0±19.3	122.0±20.3	126.0±20.7
DBP, mmHg	76.9±10.3	77.5±10.4	77.9±10.6	78.8±10.8	79.7±10.0
BMI, kg/m^2^	23.3±3.4	23.7±3.4	24.1±3.6	24.6±3.6	25.3±3.8
TG, mmol/L	1.1±0.8	1.2±0.8	1.3±0.9	1.4±1.0	1.7±1.2
TC, mmol/L	4.8±1.0	4.8±1.0	4.8±1.0	5.0±1.0	5.1±1.1
HDL-C, mmol/L	1.5±0.4	1.6±0.4	1.6±0.4	1.6±0.4	1.6±0.4
LDL-C, mmol/L	2.1±1.0	2.1±0.9	2.2±0.8	2.3±0.8	2.2±0.8
FBG, mmol/L	4.7±0.5	4.8±0.5	4.8±0.5	4.8±0.5	4.7±0.5
Log CRP*, mg/L	-0.6±1.6	-0.6±1.5	-0.5±1.5	-0.3±1.4	0.1±1.4
Medical history					
Hypertension, %	4.3	5.4	6.9	9.3	19.8
Use of Antihypertensives, %	3.6	4.8	6.1	8.3	18.7
Hyperlipidemia, %	2.5	2.9	4.3	5.7	12.1
Use of Antihyperlipidemia, %	0.3	0.3	0.5	0.8	2.8
Smoking					
never, %	98.4	98.8	98.0	98.1	96.9
former, %	0.2	0.3	0.2	0.3	0.6
current, %	1.3	0.9	1.8	1.6	2.6
Alcohol drinking					
never, %	93.0	93.7	92.4	91.9	91.0
former, %	0.5	0.4	0.3	0.5	0.5
current, %	6.5	5.9	7.3	7.6	8.5
**Men**					
No. of patient	8117	8290	8297	8265	8381
Uric acid, µmol/L	<233.9	234.0-275.0	275.0-312.9	313.0-362.0	>362.0
Uric acid Median, µmol/L	207.6	256.0	293.0	334.0	403.0
Age, year	49.3±11.8	49.7±12.0	50.0±12.4	49.8±12.8	50.6±13.5
SBP, mmHg	129.0±18.7	128.0±19.2	129.0±19.6	129.0±19.6	131.0±20.1
DBP, mmHg	83.2±11.0	82.8±11.2	83.2±11.4	83.2±11.4	84.7±11.7
BMI, kg/m^2^	24.3±3.3	24.3±3.2	24.3±3.3	25.1±3.3	25.9±3.4
TG, mmol/L	1.5±1.1	1.5±1.1	1.5±1.2	1.6±1.3	2.0±1.5
TC, mmol/L	4.6±1.2	4.8±1.1	4.9±1.0	5.0±1.1	5.1±1.1
HDL-C, mmol/L	1.6±0.4	1.6±0.4	1.5±0.4	1.5±0.4	1.5±0.4
LDL-C, mmol/L	2.3±0.9	2.3±0.9	2.3±0.9	2.4±0.8	2.3±0.9
FBG, mmol/L	4.8±0.5	4.8±0.5	4.8±0.5	4.8±0.5	4.8±0.5
Log CRP*, mg/L	-0.5±1.7	-0.5±1.6	-0.4±1.5	-0.3±1.7	-0.1±1.4
Medical history					
Hypertension, %	4.9	6.6	8.7	10.1	16.2
Use of Antihypertensives, %	4.0	5.5	7.4	8.8	14.5
Hyperlipidemia, %	2.2	2.6	3.8	4.7	7.4
Use of Antihyperlipidemia, %	0.3	0.3	0.5	0.6	1.0
Smoking					
never, %	60.8	52.7	49.7	44.8	40.2
former, %	4.4	5.2	5.9	6.9	8.6
current, %	34.8	42.0	44.4	48.3	51.2
Alcohol drinking					
never, %	62.5	55.2	49.5	42.9	35.3
former, %	3.0	3.5	4.0	3.9	4.5
current, %	34.5	41.3	46.5	53.2	60.2

^*^ Logarithmic transformed values for hsCRP.

**Table 2:** Hazard ratios (HRs) and 95% confidence interval (CI) for risk of new onset Impaired Fasting Glucose according to serum uric acid quintile among individuals in the Kailuan Study in 2006-2010.

	Uric acid quintile				
	Quintile 1	Quintile 2	Quintile3	Quintile4	Quintile5
**Women**					
Case, n	547(20.7%)	577(21.5%)	611(23.0%)	668(25.3%)	812(30.0%)
Model 1	0.97(0.86-1.10)	1.00	1.03(0.91-1.15)	1.08(0.96-1.21)	1.10(0.98-1.22)
Model 2	0.99(0.88-1.12)	1.00	0.96(0.85-1.08)	1.03(0.91-1.15)	1.02(0.91-1.14)
Model 3	0.99(0.87-1.11)	1.00	0.97(0.86-1.09)	1.03(0.92-1.15)	1.02(0.91-1.14)
**Men**					
Case, n	2617(32.2%)	2482(29.9%)	2666(32.1%)	2804(33.9%)	2982(35.6%)
Model 1	1.11(1.06-1.18)	1.00	1.07(1.00-1.10)	1.12(1.06-1.18)	1.12(1.06-1.18)
Model 2	1.11(1.05-1.17)	1.00	1.04(0.98-1.10)	1.07(1.02-1.14)	1.03(0.98-1.09)
Model 3	1.11(1.05-1.17)	1.00	1.03(0.98-1.09)	1.07(1.01-1.13)	1.02(0.97-1.08)

Note: Model 1, adjusted for age (year).

Model 2, adjusted for age (year), SBP(mmHg), DBP(mmHg), BMI(kg/m^2^), TG(mmol/L), TC(mmol/L), HDL-C(mmol/L), LDL-C(mmol/L), FBG(mmol/L), log CRP(mg/L), hypertension(yes/no), use of antihypertensives(yes/no, including diuretics, beta-blockers, alpha-blockers, angiotensin-converting enzyme inhibitors, calcium channel blockers, and angiotensin II receptor blockers), hyperlipidemia(yes/no), and use of Antihyperlipidemia (yes/no).

Model 3, adjusted for age (year), SBP(mmHg), DBP(mmHg), BMI(kg/m^2^), TG(mmol/L), TC(mmol/L), HDL-C(mmol/L), LDL-C(mmol/L), FBG(mmol/L), log CRP(mg/L), hypertension(yes/no), use of antihypertensives(yes/no, including diuretics, beta-blockers, alpha-blockers, angiotensin-converting enzyme inhibitors, calcium channel blockers, and angiotensin II receptor blockers), hyperlipidemia(yes/no), use of antihyperlipidemia (yes/no), smoking (never/former/current), and alcohol drinking (never/former/current).

To evaluate the effects of hypertension and hyperlipidemia on the incidence of new onset IFG, in the further analysis we excluded participants (8286 women and 19181 men) with hypertension (SBP≥140mmHg, DBP≥90mmHg) and hyperlipidemia (TG≥2.26mmol/L, TC≥6.22mmol/L, LDL-C≥4.14mmol/L). The overall results showed that in population excluding individuals with hypertension and hyperlipidemia there is an association between SUA and cumulative incidence of IFG (Table S1), which is in line with the results including all participants (Table S1). To clarify the potential effects of wide age range on the analysis, we stratified the participants by different age groups (<45, 45-64, and >65 years old). Cox regression analyses revealed that there is no obvious association between SUA and new onset IFG in women. In men, in different age groups there are different correlations between SUA and new onset IFG. The overall results from different age groups are similar to the results from analyses treating all ages as one group, which shows that both high and low SUA can increase the risk of new onset IFG (Table S2).

## Conclusions

One strength of this report is the large sample size, which is 185,658 person-years. Furthermore, in this report data from male and female participants are analyzed separately and many factors which are known to affect glucose metabolism are taken into consideration. There are significant differences in different genders on the association between SUA and the cumulative incidence of IFG. The major novel finding is that after adjusted to many factors which affect glucose metabolism, in male participants the correlation between SUA levels and cumulative incidence of IFG shows an U shape curve, with both low and high SUA associated with higher IFG rates. In female participants although the cumulative incidence of IFG increases with higher level of SUA, there is no significant correlation after adjusted to other factors.

Although epidemiological survey already demonstrated that the levels of SUA are significantly different in men and in women [2], few studies on SUA analyzed men and women separately. A study based on data from a health survey of Japanese population showed that elevated SUA is an independent risk factor only for women [21]. Chou et al found that SUA levels are associated with insulin resistance and plasma glucose levels more strongly in women than in men in a community based epidemiological study [22]. However, surveys of Japanese men reached different conclusions [10,14]. Three population-based studies reached the same conclusion that that SUA is an independent risk factor for diabetes [11-13]. But these studies did not analyze men and women separately. In this report, we found that men have higher level of SUA than women. Furthermore, we found that the association between SUA and abnormal glucose metabolism is different in men or women. In men different levels of SUA are associated with different cumulative incidence of IFG. In women there is no obvious association between SUA and abnormal glucose metabolism. The reasons for the differences between men and women could come from different levels of sex hormones. We also noticed that the level of SUA in the population we surveyed is lower than other reports, especially in female participants of this survey. The lower level of SUA of this report could be the partial reason that our results are different from some other studies. In summary, SUA can affect glucose metabolism in men or women differentially and the mechanisms of this difference needs further study.

The mechanism of how SUA affects glucose metabolism is unknown. Previous study showed that insulin resistant will decrease urinary uric acid clearance. Therefore high SUA could be an indicator of insulin resistant [23]. The study by Perticone et al [24] demonstrates that in essential hypertension patients, individuals with normal glucose tolerance but with high 1-hour postload glucose, individuals with abnormal glucose tolerance, as well as diabetic patients all have higher SUA, insulin resistant, and elevated hs-CRP. It suggests that high SUA could cause insulin resistant and further affect glucose metabolism [24]. In addition, SUA also has a bi-directional effect on oxidative stress. Uric acid possesses both antioxidant and pro-oxidant properties [31]. On one hand, SUA may enhance oxidative stress and inflammation and have adverse effects on glucose metabolism [25], but on the other hand, SUA is also an important component of the anti-oxidative system in human body [26]. This may be the reason why in our report the second quintile in men has the lowest rate of IFG and both higher and lower SUA can increase the rates of IFG. In cellular level, hyperuricemia can cause oxidative damage of pancreatic beta cells [32]. Previously we had a similar observation about the rate of stroke in diabetic patients in Kailuan study (unpublished results). This conclusion is also supported by publications from other groups [27,28]. It has been reported that intracellular uric acid can induce oxidative stress [29]. However, whether the level of extra cellular uric acid can reflect overall oxidative stress needs further investigation.

In summary, the data of our report are based on health examination of the Kailuan Community. This survey has the characteristic of large sample size and including different genders, age, and different types of jobs. The data can be seen as representative of population in an industrial city of north China. One limitation of this study is that we did not monitor postprandial blood glucose; therefore we may miss some diabetic patients. In the future, we plan to follow up the same population and monitor any abnormal glucose metabolism.

## Supporting Information

Table S1 **Hazard ratios (HRs) and 95% confidence interval (CI) for risk of new onset Impaired Fasting Glucose according to serum uric acid quintile among individuals in the Kailuan Study in 2006-2010 after excluding hyperlipidemia and hypertension.**
(DOC)

Table S2 **Hazard ratios (HRs) and 95% confidence interval (CI) for risk of new onset Impaired Fasting Glucose according to serum uric acid quintile among individuals in the Kailuan Study in 2006-2010 stratified by age.**
(DOC)
