# Supplementary material for: Serum Uric Acid Levels and the Risk of Impaired Fasting Glucose: A Prospective Study in Adults of North China
Source: PLoS One. 2013 Dec 23;8(12):e84712. doi: 10.1371/journal.pone.0084712 (PMC3871632; doi:10.1371/journal.pone.0084712)
Supplement: Table S1 — Hazard ratios (HRs) and 95% confidence interval (CI) for risk of new onset Impaired Fasting Glucose according to serum uric acid quintile among individuals in the Kailuan Study in 2006-2010 after excluding hyperlipidemia and hypertension. (DOC) [file pone.0084712.s001.doc]

**Table S1. Hazard ratios (HRs) and 95% confidence interval (CI) for risk of new onset Impaired Fasting Glucose according to serum uric acid quintile among individuals in the Kailuan Study in 2006-2010 after excluding hyperlipidemia and hypertension**

|  | Uric acid quintile | | | | |
| --- | --- | --- | --- | --- | --- |
|  | Quintile 1 | Quintile 2 | Quintile3 | Quintile4 | Quintile5 |
| **Women** |  |  |  |  |  |
| Case, n | 295 (17.9%) | 303 (18.5%) | 320 (19.3%) | 325 (19.6%) | 391 (23.2%) |
| Model 1 | 0.98 (0.83-1.15) | 1.00 | 1.034 (0.88-1.21) | 0.98 (0.84-1.15) | 1.03 (0.89-1.20) |
| Model 2 | 0.99 (0.84-1.16) | 1.00 | 0.96 (0.82-1.13) | 0.92 (0.78-1.07) | 0.93 (0.80-1.09) |
| Model 3 | 0.98 (0.84-1.16) | 1.00 | 0.96 (0.82-1.13) | 0.92 (0.79-1.08) | 0.92 (0.79-1.08) |
| **Men** |  |  |  |  |  |
| Case, n | 1150 (30.8%) | 1057 (27.2%) | 1143 (29.6%) | 1106 (28.9%) | 1203 (31.1%) |
| Model 1 | 1.19 (1.09-1.29) | 1.00 | 1.11 (1.02-1.21) | 1.06 (0.98-1.16) | 1.08 (0.99-1.17) |
| Model 2 | 1.18(1.08-1.28) | 1.00 | 1.10 (1.01-1.19) | 1.05 (0.96-1.14) | 1.06 (0.98-1.16) |
| Model 3 | 1.19 (1.09-1.29) | 1.00 | 1.09 (1.00-1.19) | 1.04 (0.96-1.14) | 1.05 (0.96-1.14) |

Note: Model 1, adjusted for age (year).

Model 2, adjusted for age (year), SBP(mmHg), DBP(mmHg), BMI(kg/m2), TG(mmol/L)， TC(mmol/L), HDL-C(mmol/L), LDL-C(mmol/L), FBG(mmol/L), log CRP(mg/L), hypertension(yes/no), use of antihypertensives(yes/no, including diuretics, beta-blockers, alpha-blockers, angiotensin-converting enzyme inhibitors, calcium channel blockers, and angiotensin II receptor blockers), hyperlipidemia(yes/no), and use of Antihyperlipidemia (yes/no).

Model 3, adjusted for age (year), SBP(mmHg), DBP(mmHg), BMI(kg/m2), TG(mmol/L)， TC(mmol/L), HDL-C(mmol/L), LDL-C(mmol/L), FBG(mmol/L), log CRP(mg/L), hypertension(yes/no), use of antihypertensives(yes/no, including diuretics, beta-blockers, alpha-blockers, angiotensin-converting enzyme inhibitors, calcium channel blockers, and angiotensin II receptor blockers), hyperlipidemia(yes/no), use of antihyperlipidemia (yes/no), smoking (never/former/current), and alcohol drinking (never/former/current).
