# Supplementary material for: Serum Uric Acid Levels and the Risk of Impaired Fasting Glucose: A Prospective Study in Adults of North China
Source: PLoS One. 2013 Dec 23;8(12):e84712. doi: 10.1371/journal.pone.0084712 (PMC3871632; doi:10.1371/journal.pone.0084712)
Supplement: Table S2 — Hazard ratios (HRs) and 95% confidence interval (CI) for risk of new onset Impaired Fasting Glucose according to serum uric acid quintile among individuals in the Kailuan Study in 2006-2010 stratified by age. (DOC) [file pone.0084712.s002.doc]

**Table S2. Hazard ratios (HRs) and 95% confidence interval (CI) for risk of new onset Impaired Fasting Glucose according to serum uric acid quintile among individuals in the Kailuan Study in 2006-2010 stratified by age**

|  | Uric acid quintile | | | | |
| --- | --- | --- | --- | --- | --- |
|  | Quintile 1 | Quintile 2 | Quintile3 | Quintile4 | Quintile5 |
| **age** | **＜45 years** | | | | |
| **Women** |  |  |  |  |  |
| Case, n | 198 (17.1%) | 209 (17.2%) | 185 (15.7%) | 210 (17.7%) | 232 (19.5%) |
| Model 1 | 0.99 (0.82-1.21) | 1.00 | 0.92 (0.75-1.12) | 0.98 (0.81-1.19) | 1.09 (0.91-1.32) |
| Model 2 | 1.00 (0.82-1.22) | 1.00 | 0.89 (0.73-1.09) | 0.94 (0.78-1.14) | 0.88 (0.72-1.07) |
| Model 3 | 0.99 (0.81-1.21) | 1.00 | 0.88 (0.72-1.08) | 0.94 (0.77-1.15) | 0.86 (0.70-1.04) |
| **Men** |  |  |  |  |  |
| Case, n | 924 (32.5%) | 810 (28.4%) | 842 (29.1%) | 840 (29.5%) | 855 (29.7%) |
| Model 1 | 1.21 (1.11-1.34) | 1.00 | 1.02(0.93-1.13) | 1.02 (0.93-1.12) | 1.00 (0.91-1.10) |
| Model 2 | 1.21 (1.10-1.33) | 1.00 | 1.02 (0.92-1.12) | 0.96 (0.87-1.06) | 0.90 (0.81-0.99) |
| Model 3 | 1.20 (1.09-1.32) | 1.00 | 1.01 (0.91-1.11) | 0.96 (0.86-1.06) | 0.88 (0.80-0.98) |
| **age** | **45-64 years** | | | | |
| **Women** |  |  |  |  |  |
| Case, n | 305 (23.3%) | 354 (27.1%) | 380 (28.9%) | 408 (30.6%) | 468 (35.5%) |
| Model 1 | 0.87(0.75-1.01) | 1.00 | 1.06 (0.92-1.23) | 1.09 (0.95-1.26) | 1.14 (0.99-1.32) |
| Model 2 | 0.88 (0.75-1.04) | 1.00 | 0.98 (0.84-1.14) | 1.05 (0.90-1.22) | 1.08 (0.93-1.26) |
| Model 3 | 0.88 (0.75-1.04) | 1.00 | 0.99 (0.85-1.15) | 1.05 (0.90-1.22) | 1.07(0.91-1.24) |
| **Men** |  |  |  |  |  |
| Case, n | 1422 (32.3%) | 1378 (30.8%) | 1471 (33.1%) | 1610 (36.4%) | 1715 (38.5%) |
| Model 1 | 1.08 (1.00-1.16) | 1.00 | 1.10 (1.02-1.18) | 1.21 (1.12-1.30) | 1.18 (1.10-1.27) |
| Model 2 | 1.07 (0.99-1.16) | 1.00 | 1.07 (0.99-1.15) | 1.17(1.09-1.26) | 1.11(1.03-1.20) |
| Model 3 | 1.08 (1.00-1.16) | 1.00 | 1.06 (0.98-1.14) | 1.17 (1.08-1.26) | 1.10(1.02-1.19) |
| **age** | **≥65 years** | | | | |
| **Women** |  |  |  |  |  |
| Case, n | 48 (29.6%) | 53 (33.1%) | 49 (29.3%) | 53 (32.3%) | 63 (38.2%) |
| Model 1 | 0.93 (0.63-1.37) | 1.00 | 0.84 (0.57-1.24) | 0.95 (0.65-1.39) | 1.02 (0.72-1.50) |
| Model 2 | 0.96 (0.63-1.46) | 1.00 | 0.96 (0.63-1.47) | 1.10 (0.70-1.72) | 1.29 (0.85-1.96) |
| Model 3 | 0.94 (0.62-1.44) | 1.00 | 0.95 (0.62-1.46) | 1.05 (0.666-1.66) | 1.25 (0.81-1.92) |
| **Men** |  |  |  |  |  |
| Case, n | 295 (30.5%) | 301 (31.3%) | 334 (34.5%) | 371 (38%) | 383 (39.4%) |
| Model 1 | 0.96 (0.82-1.13) | 1.00 | 1.07 (0.91-1.25) | 1.16 (1.00-1.35) | 1.22 (1.05-1.42) |
| Model 2 | 0.90 (0.76-1.06) | 1.00 | 0.96 (0.81-1.13) | 1.13 (0.96-1.32) | 1.13 (0.96-1.33) |
| Model 3 | 0.89 (0.75-1.06) | 1.00 | 0.95 (0.81-1.12) | 1.16 (0.95-1.31) | 1.15 (0.97-1.36) |

Note: Model 1, adjusted for age (year).

Model 2, adjusted for age (year), SBP (mmHg), DBP(mmHg), BMI(kg/m2), TG(mmol/L)， TC(mmol/L), HDL-C(mmol/L), LDL-C(mmol/L), FBG(mmol/L), log CRP(mg/L), hypertension(yes/no), use of antihypertensives(yes/no, including diuretics, beta-blockers, alpha-blockers, angiotensin-converting enzyme inhibitors, calcium channel blockers, and angiotensin II receptor blockers), hyperlipidemia(yes/no), and use of Antihyperlipidemia (yes/no).

Model 3, adjusted for age (year), SBP (mmHg), DBP(mmHg), BMI(kg/m2), TG(mmol/L)， TC(mmol/L), HDL-C(mmol/L), LDL-C(mmol/L), FBG(mmol/L), log CRP(mg/L), hypertension(yes/no), use of antihypertensives(yes/no, including diuretics, beta-blockers, alpha-blockers, angiotensin-converting enzyme inhibitors, calcium channel blockers, and angiotensin II receptor blockers), hyperlipidemia(yes/no), use of antihyperlipidemia (yes/no), smoking (never/former/current), and alcohol drinking (never/former/current).
